# Advances in Solution-Processed Blue Quantum Dot Light-Emitting Diodes

**DOI:** 10.3390/nano13101695

**Published:** 2023-05-22

**Authors:** Sheng-Nan Li, Jia-Lin Pan, Yan-Jun Yu, Feng Zhao, Ya-Kun Wang, Liang-Sheng Liao

**Affiliations:** 1 Institute of Functional Nano & Soft Materials (FUNSOM), Jiangsu Key Laboratory for Carbon-Based Functional Materials & Devices, Soochow University, Suzhou 215123, China; lishengnan1004@163.com (S.-N.L.); jlpan1013@163.com (J.-L.P.); yuyanjun1998@163.com (Y.-J.Y.); fengzhao_lum@163.com (F.Z.); 2 Macao Institute of Materials Science and Engineering, Macau University of Science and Technology, Taipa 999078, Macau SAR, China

**Keywords:** blue QLED, II-V QD, III-V QD, carbon dot, perovskite QD

## Abstract

Quantum dot light-emitting diodes (QLEDs) have been identified as a next-generation display technology owing to their low-cost manufacturing, wide color gamut, and electrically driven self-emission properties. However, the efficiency and stability of blue QLEDs still pose a significant challenge, limiting their production and potential application. This review aims to analyse the factors leading to the failure of blue QLEDs and presents a roadmap to accelerate their development based on the progress made in the synthesis of II-VI (CdSe, ZnSe) quantum dots (QDs), III-V (InP) QDs, carbon dots, and perovskite QDs. The proposed analysis will include discussions on material synthesis, core-shell structures, ligand interactions, and device fabrication, providing a comprehensive overview of these materials and their development.

## 1. Introduction

Quantum dots (QDs) are an important low-dimensional semiconductor material characterized by dimensions that are not larger than twice the exciton Bohr radius of their corresponding pairs of bulk materials. The low-dimensional structure of QDs enables electron-hole radiative recombination by binding electrons and holes in a small space. Additionally, QDs can emit in a wide range, from visible to near-infrared, through the quantum confinement effect (QCE). These advantages make II-VI (CdSe, ZnSe), III-V (InP), and IV (Carbon dot) QDs attractive for various applications.

Among different kinds of QDs, CdSe has a photoluminescence quantum yield (PLQY) close to 100% and excellent photostability, making Cd-based light-emitting diodes highly efficient, with an external quantum efficiency (EQE) exceeding 20% [1]. However, the toxicity of Cd-based materials limits their commercial applications. Therefore, researchers have focused on developing heavy metal-free QDs, such as InP, ZnSe, and carbon dots (CDs). InP has a bandgap similar to that of CdSe but has a larger exciton Bohr radius. Although CDs are not suitable for display due to impurities and defect states, they show promise in bioimaging applications due to their good biocompatibility. The new perovskite QDs (PQDs) exhibit high color purity and PLQY. Additionally, PQD emission can be tuned through QCE and halogen composition, making them an attractive material for various applications.

QD light-emitting diodes (QLEDs) unite the advantages of low cost, solution processing, and low-temperature fabrication and production, which makes them stand out among other emitters [2,3,4]. Theoretically, QLEDs can achieve ultrahigh color purity, with the National Television System Committee standard of 140% [5].

Compared to red and green QLED devices, blue QLEDs have lower performance. For instance, using InP QLEDs as an example, blue InP QLEDs exhibit a luminance of <5000 cd m^−2^ and a maximum EQE of < 3% [6]. In contrast, red InP QLEDs can achieve an EQE, luminance, and T_95_ (the time for the brightness to drop to 95% of its initial value) of 22.2%, >110,000 cd m^−2^, and >32,000 h, respectively [7]. Similarly, a green InP QLED can attain an EQE, luminance, and T_50_ (the time for the brightness to drop to 50% of its initial value) of 16.3%, >12,000 cd m^−2^, and >1000 h, respectively [8]. Until now, red and green QLEDs have already satisfied the requirements for commercial applications, whereas blue QLEDs still have a long way to go. The reasons for this disparity include the fact that 1. blue QDs necessitate small particle sizes, which results in difficulties in synthesizing uniform QDs, and 2. the wide bandgap that makes exciton traps sensitive and results in nonradiative recombination. The synthesis methods for various types of QDs exhibit similarities. For example, Peng et al. synthesized InP QDs at low reaction temperatures by utilizing aliphatic amine ligands, which is a technique borrowed from the synthesis of II-VI QDs [9]. Similarly, Jang et al. utilized HF etching to prepare high-efficiency blue ZnTeSe QLEDs, achieving ZnSeTe QLEDs with a luminance of 88,900 cd m^−2^, an EQE of 20.2%, and a T_50_ time of 15,850 h at 100 cd m^−2^ [10]. Thus, a comprehensive analysis of the synthetic methodology, surface ligand treatment, and device engineering of blue QDs is necessary for the advancement of blue QLEDs.

This review aims to provide an overview of blue QLEDs using II-VI QDs, III-V QDs, CDs, and PQDs. In Section 2, we introduce the properties, nucleation, and growth mechanisms of the aforementioned QD materials, as well as the principle of electroluminescence (EL) of QDs. Section 3 and Section 4 focus on the synthesis and device engineering of II-VI (CdSe, ZnSe) and III-V (InP) inorganic QDs. In Section 5, we discuss the photoluminescence (PL) and EL prospects of CDs. Finally, in Section 6, we introduce newly developed PQDs from both QCE and mixed halogen perspectives. We hope that this review will provide researchers with a better understanding of the strengths and weaknesses of each type of QD, as well as the next steps for further improving the performance of blue QLEDs.

## 2. Properties of QDs

### 2.1. Quantum Confinement Effect

**Figure 1:**
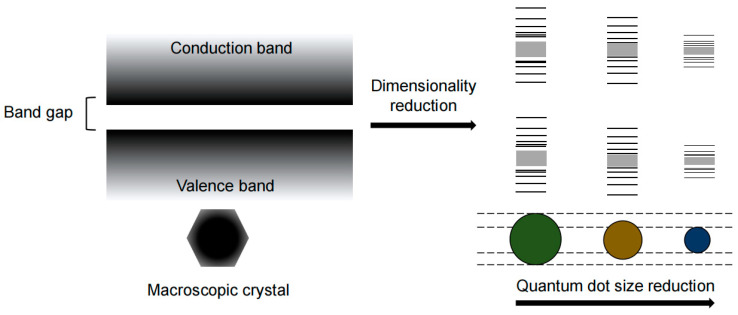
Schematic of QCE under different QD sizes.

QCE is a phenomenon where the energy levels of a material become discrete when the particle is close to or smaller than the Bohr radius of the exciton (Figure 1). This phenomenon allows for the manipulation of the physical properties of semiconductor nanocrystals (NCs) by controlling their size. QDs are a type of NC, and their size can be precisely controlled, enabling tuning of their emission light from ultraviolet to infrared, which is a practical application of QCE [11].

### 2.2. Growth and Nucleation Mechanism of QDs

The minimization of Gibbs free energy is the chemical driving force for QD nucleation during synthesis. In the absence of a kinetic energy barrier, the total surface free energy of a crystallization system includes the area of each section (*A_i_*) of each crystal in the solution and is related to the specific surface energy (*σ_i_*). The relationship is shown in Equation (1).

(1) $$\sum G_{surface}=\sum \sigma_{i}A_{i}\approx \sigma \sum A_{i}=minimum$$

The dangling bonds carried by the atoms on the crystal surface are the direct source of the crystal surface free energy. The surface Gibbs free energy decreases as the grain size increases. In addition to natural growth, appropriate ligands or specific solvent molecules can also reduce the number of dangling bonds [12].

The surface Gibbs free energy of NCs in Equation (1) decreases as size increases because of the reduced surface-to-volume atomic ratio. Therefore, the solubility of NCs decreases drastically as their size increases, which comes from the Gibbs-Thompson equation (Equation (2))

(2) $$S_{d}=S_{\infty }exp\left(4\sigma V_{m}/dRT\right)$$

*σ* and *V_m_* are the specific surface free energy and molar volume of the crystal, respectively, *S_∞_* is the solubility of the blocky crystal, *S_d_* is the solubility of the crystal with diameter *d*, *R* is the gas constant, and *T* is the absolute temperature. In the process of NC nucleation and growth, larger crystals grow at the expense of smaller crystals due to the difference in surface energy between them, leading to the “size distribution out of focus” of NCs in the system [13]. This is because the smaller particles have a higher surface energy and tend to dissolve into the solution, while the larger particles have a lower surface energy and tend to absorb more monomers from the solution and grow further. This is the Oswald ripening process.

When the size of the studied NCs changes from the nanoscale to the micron scale, the surface-to-atom ratio decreases rapidly with increasing crystal size. This leads to the fact that the surface energy in the crystal is negligible compared to the total free energy of the system. Therefore, new methods are needed to study the crystallization process of NCs at the nanoscale. Peng’s group used a computer to deconvolve the absorption spectrum to obtain QD information on the size distribution. The method mentioned in the previous statement is known as the “deconvolution method”, which can be used to obtain the size distribution of QDs from their absorption spectra. Briefly, the ultraviolet–visible (UV–vis) absorption spectra of the samples were deconvoluted to obtain a series of contributing factors for the standard spectra. The corresponding particle size distribution profile can be obtained by adding the products of each contributing factor and the corresponding Gaussian particle size distribution of the standard sample [14].

### 2.3. Charge Transport and EL Properties of QDs

**Figure 2:**
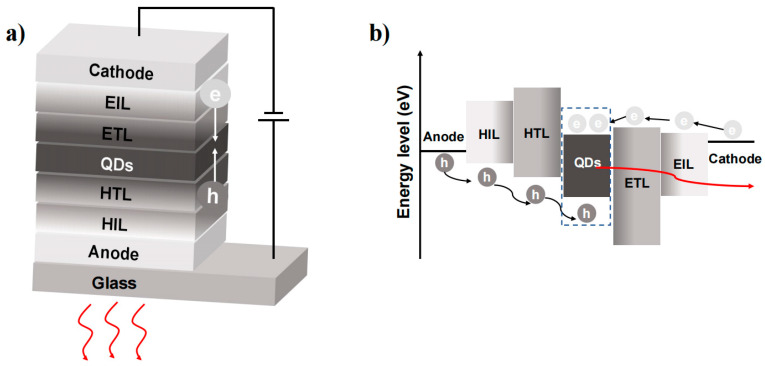
(**a**) The traditional structure of QLEDs. (**b**) Schematic of the processes of charge injection and charge recombination in an energy band diagram.

A typical QLED architecture is shown in Figure 2a, which contains an anode, a hole-injecting layer (HIL), a hole-transporting layer (HTL), a light-emitting layer (EML), an electron-transporting layer (ETL), an electron-injecting layer (EIL) and a cathode. The EL of QDs is generated by the radiative recombination of holes and electrons through the EML. QDs with long-chain ligands have insulating properties and make carrier injection difficult. To overcome this challenge and improve carrier injection efficiency, QDs require surface modification or ligand exchange with short chains. Furthermore, from a device perspective, the EML needs a HIL and EIL that reside between the electrode and the EML.

The implementation of a high mobility hole (electron) transporting layer (HTL/ETL) helps facilitate carrier recombination. However, the injection of electrons and holes into the EML does not always occur simultaneously. The conduction band shifts of the EML and ETL are typically much smaller than the valence band shifts of the EML and HTL. This discrepancy results in a build-up of excess electrons within the EML, leading to negatively charged QDs that are unable to undergo radial excitation (Figure 2b). Moreover, the accumulation of excess electrons can leak into the hole-transporting layers and cause a decrease in the device’s lifetime [15,16]. Therefore, the fundamental principle in designing high-performance QLEDs is to focus on balancing charge injection and effective radiative recombination.

## 3. Ⅱ-Ⅵ QDs

### 3.1. Cd-Based QDs

(1) Synthesis and modification of CdSe QDs

**Figure 3:**
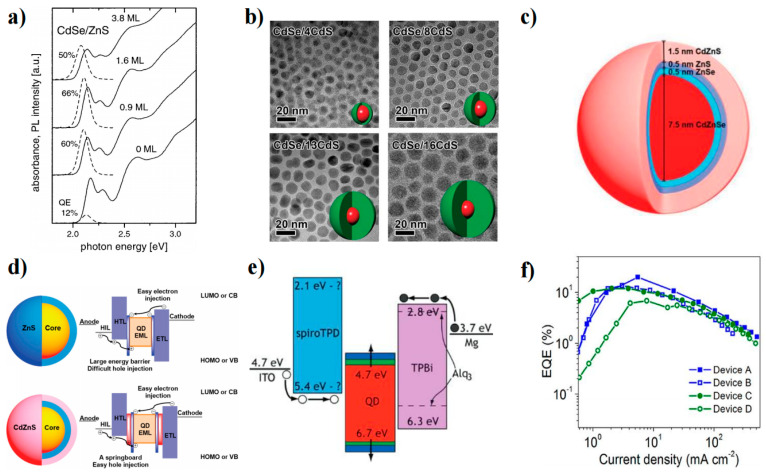
(**a**) UV–vis absorption (solid lines) and PL spectra (dashed lines) of CdSe QDs before and after deposition of ZnS shells of different thicknesses [20]. Copyright 2001, American Chemical Society. (**b**) TEM images of CdSe/xCdS QDs [25]. Copyright 2012, American Chemical Society. (**c**) The corresponding core/shell structure of CdZnSe/ZnSeS/ZnS/CdZnS QDs [26]. Copyright 2022, Wiley-VCH. (**d**) Multishell structure QD and an energy diagram in a conventional QLED [26]. Copyright 2022, Wiley-VCH. (**e**) All-organic QLED device structures of the energy diagram [27]. Copyright 2009, American Chemical Society. (**f**) EQE—current density curve of the blue CdSe/ZnS QLED [28]. Copyright 2017, American Chemical Society.

CdSe (1.74 eV), a direct bandgap semiconductor, has attracted extensive attention and in-depth study in the early stages of research [17,18,19]. The prenucleation of CdSe QDs is commonly achieved using the hot injection method. The injection of metal precursor solutions into different ligand systems can have a significant impact on the growth kinetics of CdSe QDs. Murray et al. utilized organometallic reagents for homogeneous nucleation by rapid injection into hot tri-n-octyl phosphine/tri-n-octyl phosphine oxide (TOP/TOPO)-liganded solvents [18]. Slow growth and annealing led to a uniform QD surface. As a result, nearly monodisperse (diameter <5% rms) samples were obtained. In another study, Talapin et al. introduced hexadecyl amine (HDA) into the TOP/TOPO system [20]. The ratio between TOPO and HDA strongly affected the growth kinetics of CdSe QDs. The initial particle size and growth rate of CdSe QDs decreased with increasing HDA content, which enabled better control of the dynamic growth of CdSe QDs. In addition, HDA not only solved the problem of the nonrepeatable shape of CdSe QDs in the TOP/TOPO system but also significantly improved the CdSe QD band-edge emitting efficiency at room temperature. The band-edge PLQY of CdSe QDs can be increased to 40–60% by surface passivation of inorganic (ZnS) or organic (alkylamine) shells (Figure 3a).

The QD core is covered by a wide-bandgap shell to reduce dangling bonds, passivate nonradiative surface sites, and improve PLQY. CdSe QD cores coated with CdS and ZnS thin shell layers can increase the PLQY by more than 50% [21]. However, a thin shell is not the perfect choice for QDs because of the loss or denaturation of ligands during solvent transfer, which will lead to incomplete passivation of surface defects and thus affect the PLQY. To address the instability of ligands in thin-shell QDs, Chen’s group developed giant shell QDs (g-QDs) [22]. The ultrathick shell completely isolated the core from the QD surface. The thick shell effectively acted as a spacer between adjacent QDs and suppressed distance-dependent interactions between particles, such as Förster resonance energy transfer [23,24]. As a result, g-QDs had strong chemical stability and high photostability in photobleaching, which improved blinking behavior (Figure 3b) [24,25]. Multiple growths of one shell layer on the core can result in the accumulation of interlayer defects. Further design of a multishell structure with alloying can release the stress caused by a lattice mismatch between the core and shell, which can result in high-quality QDs with low defect densities. Moreover, energy level matching between the QDs and the transport layer can be realized by adjusting the composition of the QDs. Liu et al. successfully designed CdZnSe/ZnSeS/ZnS/CdZnS QDs (Figure 3c) to achieve efficient blue emission [26]. On the condition that the CdZnSe/ZnSeS/ZnS double shell structure enables high PLQY, a layer of CdZnS with a narrow band gap is added to promote the holes that inject the EML (Figure 3d).

(2) Blue CdSe-based QLEDs

Since the first report of CdSe QLEDs in 1994, a series of explorations on on-device application and synthesis have been developed [17,29,30,31,32,33]. Initially, the device structure was simple, where QDs were used as both the emissive and transport layers, resulting in poor PLQY [17]. As OLED devices progressed, researchers borrowed the transport layer design and tried to prepare all-organic devices by inserting QDs between two organic transport layers (Figure 3e) [27]. However, this structure is not ideal due to concerns about the stability of organic devices. While all-inorganic devices exhibit greater thermal and chemical stability, there are still issues with inorganic metal oxides, such as complex surface defects and a lack of research on the physical mechanism of exciton formation. Currently, the most widely used and efficient devices are organic–inorganic hybrid devices, where organic compounds (such as PEDOT:PSS, poly-TPD, PVK, and TFB) are used in the HIL and HTL, while inorganic metal oxides (such as ZnO and ZnMgO) are used in the ETL and the EML is placed in the middle position. Various typical structures were summarized in Qi’s article [19,34]. Over more than a decade of development, the performance of blue QLEDs has rapidly improved. At present, the Cd-based blue QLED performance can reach EQE = 19.8%, maximum luminance (L_max_) = 62,600 cd m^−2^, and T_50_ > 10,000 h (Figure 3f). Qian et al. introduced ZnO as an ETL and fabricated blue QLEDs with an EQE of 0.22% by using an organic–inorganic hybrid structure [29]. Subsequently, Wang et al. increased the EQE of blue QLEDs to 19.8% (excluding tandem devices; the EQE for current tandem blue QLEDs is 24%) [28,35,36,37,38,39,40,41,42]. During this period of rapid development, blue QLED performance was optimized from three aspects (Table 1): (1) core-shell structure and ligand engineering to reduce nonradiative recombination (Auger recombination and Förster resonance energy transfer, etc.), (2) device interface engineering to avoid interlayer charge accumulation, and (3) the electron-blocking layer to balance the charge and promote radiation recombination due to the deep HOMO level of blue QDs, making hole injection more difficult than electron injection.

### 3.2. ZnSe-Based QDs

(1) Synthesis and modification of ZnSe-based QDs

**Figure 4:**
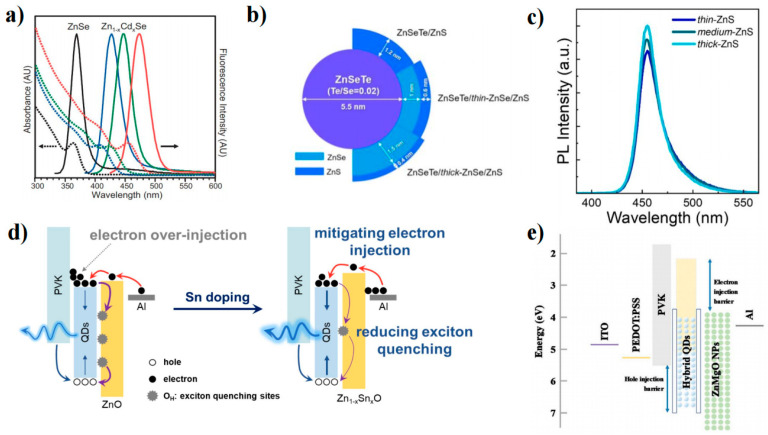
(**a**) UV–vis absorption (dashed lines) and PL spectra (solid lines) of ZnSe and Zn_1–x_Cd_x_Se alloyed QDs [48]. Copyright 2007, Wiley-VCH. (**b**) Schematic of ZnSeTe core to double shells of ZnSeTe/thin-, medium-, and thick-ZnSe/ZnS C/S/S QDs [53]. Copyright 2017, American Chemical Society. (**c**) PL spectra of ZnS outer shell thickness-dependent C/S/S QDs [54]. Copyright 2021, Elsevier B. V (**d**) Carrier injection and recombination mechanism for these deep-blue-emitting devices [55]. Copyright 2022, American Chemical Society. (**e**) Device and energy level structure of QLEDs with hybrid QDs [56]. Copyright 2020, Wiley-VCH.

ZnSe has emerged as a promising alternative to Cd-based materials in the blue region due to its nontoxicity and appropriate bandgap (2.7 eV). Margaret’s group studied ZnSe QD growth and investigated how to control the properties of the QDs [45]. They synthesized monodisperse and highly luminescent ZnSe QDs in an HDA/TOPO coordination solvent and achieved tunable band-edge fluorescence between 2.8 and 3.4 eV at room temperature. Moreover, high-brightness ZnSe QDs with a PLQY of 72% and high reproducibility were obtained by non-injection [46]. Yu et al. synthesized ZnSe QDs with uniform size and controlled shape by heating a mixture of Zn(Ac)_2_, Se powder, oleic acid, and liquid paraffin instead of direct injection of the Se source. This method avoided the use of dangerous and unstable alkyl phosphines, such as TOPO [47]. Zheng et al. developed water-synthesized glutathione-capped ZnSe and Zn_1-x_Cd_x_Se alloy QDs with adjustable fluorescence emission between 360 and 500 nm and a high PLQY of 50% (Figure 4a) [48]. This is also the first study to directly synthesize blue QDs in an aqueous solution. Although element doping can adjust the ZnSe bandgap, nontoxic elements such as Te, Mn, and Cu are more suitable for doping than Cd [49,50]. The optimal choice for achieving ZnSe QD blue light emission is to form an alloy with ZnTe (2.25 eV). Lesnyak et al. proposed a simple one-step water synthesis of glutathione covering ZnSe_1−x_Te_x_ QDs, which avoided the complexity of multistage preparation and achieved a PLQY of 20% [47].

ZnSe and ZnS are commonly used as shell layers to improve the PLQY and brightness of QDs. Dong et al. proposed a two-step synthesis method to synthesize ZnSe/ZnS QDs in the 390–460 nm range under mild conditions (150 °C) [51]. Recently, Yang’s group also reported a seed-mediated double-shell strategy in which two ZnS shells were prepared by two different shell-forming steps to obtain ZnSe/ZnS/ZnS core-shell QDs with high PLQY and good stability. Lad et al. synthesized ZnSe and ZnSe/ZnS QDs by a high-temperature wet chemical route and found four excited states (1S^e^ − 1S_3/2_, ${1S}^{e}{-1S}_{\frac{3}{2}}^{h}$, ${1P}^{e}{-1P}_{\frac{3}{2}}^{h}$, and 1S^e^ − 1S^SO^) [52]. The four excited states vary with the ZnS shell layer thickness, which can visually reflect how the electronic energy level of the ZnSe QD changes.

(2) Blue ZnSe-based QLEDs

Jang et al. investigated the synthesis of binary and ternary ZnSe-based QDs. Specifically, they explored the effects of increasing the thickness of the ZnSe shell and adjusting the Se/Te ratio in ZnSeTe on the performance of the blue QLED (Figure 4b). To further enhance device performance, ZnSe was introduced between the ZnSeTe and ZnS interfaces, resulting in the successful fabrication of high-performance blue ZnSeTe/ZnSe/ZnS QLEDs. Furthermore, Lee et al. adopted a two-step method to synthesize ZnSeTe/ZnSe/ZnS QDs and tuned the thickness of the ZnSe inner shell and the ZnS shell to investigate the effect on the performance of blue QLEDs (Figure 4c). Their results indicated that the thickness modulation of the ZnSe inner shell not only influenced the growth of the ZnS shell but also affected the peak wavelength, color purity, and PLQY of the QDs. Additionally, as the thickness of the ZnS shell increased, the injection of electrons was found to be more effectively suppressed relative to holes, resulting in improved charge balance and enhanced EQE.

To improve the efficiency and stability of blue ZnSe-based QLEDs, researchers have also explored various aspects, such as device structures, surface passivation, material preparation, and simulation of ZnSe-based QDs (Table 2) [57]. Currently, there are two main approaches to developing ZnSe-based QLEDs: on the one hand, the EML is doped to mitigate the electron leakage current, which is caused by the high intrinsic electron mobility of ZnSe QDs; on the other hand, the ETL is doped to regulate the energy band and reduce the electron mobility while suppressing the surface defect states. Cho et al. utilized P-type semiconductors (TCTA and TPD) to dope ZnSe (Figure 4e) [56]. Hybrid QDs were found to effectively prevent electron overflow from the hole transport materials and improve the hole current characteristics due to the p-type doping effect. ZnO is commonly used as the ETL in blue-light devices. Efforts have been made to optimize the physical properties of ZnO NPs by doping elements such as Mg, Li, Cl, S, Al, and Ga [58,59,60,61,62,63,64]. Gao et al. reported the use of Sn-doped ZnO, which not only reduced the exciton quenching sites on the metal oxide surface caused by OH but also decreased the electron mobility and slowed down the over injection of electrons (Figure 4d) [55]. Kim et al. modulated the emission wavelength of ZnSeTe to 457 nm by adding Te. They fabricated a double-QD EML with graded chloride content in the LED to facilitate hole transport. The resulting device has high efficiency (EQE = 20.2%), high brightness (L_max_ = 88,900 cd m^−2^), and a long operating life (T_50_ = 15,850 h at 100 cd m^−2^) [10]. This is the best performance of an inorganic blue QLED at present.

## 4. Ⅲ-Ⅴ QDs

### 4.1. Development of InP QD Synthesis

**Figure 5:**
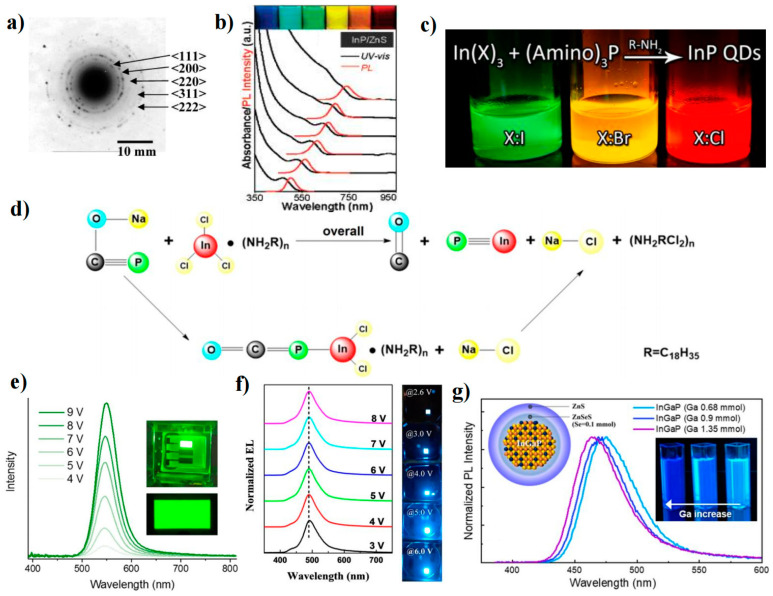
(**a**) HRTEM image of an InP QD [74]. Copyright 2005, American Chemical Society. (**b**) UV–vis absorption and PL spectra of different sized InP/ZnS core/shell QDs [9]. Copyright 2007, American Chemical Society. (**c**) Schematic of the synthesis of InP QDs from indium halide and aminophosphine precursors [78]. Copyright 2015, American Chemical Society. (**d**) Schematic for the formation of InP QDs by using InCl_3_ and NaOCP [79]. Copyright 2021, American Chemical Society. (**e**) Voltage-dependent EL spectra of the green QLED [8]. Copyright 2017, Nature Publishing Group. (**f**) EL spectra at different voltages [80]. Copyright 2020, American Chemical Society. (**g**) Normalized PL spectra of InGaP QDs [81]. Copyright 2020, American Chemical Society.

InP QDs are promising candidates for heavy-metal-free QDs because of their wider spectral range, narrow PL linewidth, and high PLQY properties, which are achieved through control of the size distribution of the InP core and engineering of the heterostructure. Previous studies on the synthesis of InP QDs aimed to obtain products with mild reaction conditions, controllable size, and high PLQY. However, the high reactivity of atoms or ions and large reaction barriers in the chemical pathways for the crystal formation of III-V QDs complicate their nucleation and growth processes. To overcome the high reaction barrier of InP QDs, researchers generally used highly reactive (SiMe_3_)_3_P as the P precursor due to its high reactivity [69,70,71]. In 1994, Nozik et al. first used InCl_3_ and (SiMe_3_)_3_P to synthesize InP QDs [70]. In 1994, Nozik et al. first used InCl_3_ and (SiMe_3_)_3_P to synthesize InP QDs. Similar to the synthesis of II-VI QDs, the concentration of ligands in a noncoordinating solvent can change the reactivity of Cd and Zn precursors, resulting in the formation of high-quality QDs [70,72]. The effect of ligand concentration on the reactivity of III-V precursors is more significant. Peng et al. reduced the InP nucleation time from days to hours by using octadecene (ODE) as a noncoordinating solvent and fatty acids as ligands [73]. Lucey et al. also synthesized InP QDs with better monodispersity in ODE by using indium carboxylate and (SiMe_3_)_3_P without surfactants or ligand solvents [74]. We can observe the lattice edges of the QD from the high-resolution transmission electron microscopy (HRTEM) of a single InP QD (Figure 5a). However, some studies suggest that a similar nucleation process in a noncoordinating solvent can also be obtained when the coordination effect of the solvent is much weaker than that of the introduced strong ligand. Xu’s group used fatty acid lipids (methyl myristate and dibutyl sebacate) as weakly coordinating solvents, as well as a more reactive indium precursor (trimethylindium) and a proton reagent to accelerate the synthesis [75]. The nucleation process produced QDs with a narrow size (2.5 nm). The results showed that high-boiling esters can act as effective, weakly coordinating solvents to control the nucleation process. Peng et al. used aliphatic amines as activating reagents to improve the reactivity of indium carboxylate and reduce the reaction temperature of the synthesis [9]. InP/ZnS QDs with controllable size distributions were successfully synthesized over a wide spectral range (450–750 nm) (Figure 5b). In addition to controlling the InP QD size to tune its emission wavelength, changing the thickness ratio between the core and shell can also perform spectral modulation. Yang et al. made the PL spectra of InP QDs cover the entire visible spectrum (350–800 nm) by changing the ratio of InP:ZnS [76].

Compared with the smaller Bohr radius (4.6 nm) of CdSe QDs, the InP system has obvious intrinsic advantages (the Bohr radius is 9.6 nm). However, the development of InP QDs in the blue region is not ideal, mainly because it is difficult to find suitable reaction conditions and precursors to control the growth of InP QDs. Shen et al. reported a colloidal approach mediated by zinc halide to improve the performance of blue-emitting InP/ZnS QDs [77]. The high reaction rate of blue InP QDs was attributed to both P and In precursors, and the use of tris(dimethylamino)phosphine ((DMA)_3_P) as the P precursor could suppress the QD size distribution (Figure 5c). Excess I^-^ was found to increase the reactivity of In^3+^ and could be used to accelerate the cleavage of In-X compared to Cl^−^ [78]. Furthermore, excess I^−^ could be combined with oleylamine (OAM) and adsorbed on the surface of InP QDs as a passivation agent, which suppressed the size distribution and reduced surface defects. Thus, an amine halide passivation layer was formed by a ZnS halide-mediated colloidal approach, leading to a PLQY of 76.1%. Due to the highly toxic and flammable nature of (DMA)_3_P, Yu et al. replaced it with the inorganic solid metal phosphorus, sodium phosphaethynolate (NaOCP), to synthesize InP QDs (Figure 5d). Inorganic solid phosphine source synthesized InP QDs with a modulable PL emission range of 465–620 nm. The PLQYs of blue (465 nm), green (533 nm), and red (620 nm) QDs were found to be 43%, 97%, and 95%, respectively [79].

### 4.2. Blue InP-Based QLEDs

In recent years, InP QLEDs have made significant progress in both red and green emissions [82,83]. Red InP QLEDs, for example, achieved an EQE of 21.4% and a luminance of >100,000 cd m^−2^ [84]. Similarly, Green InP QLEDs have demonstrated an EQE of 16.3% after passivation with different alkyl diamines and zinc halides (Figure 5e) [85]. Despite these successes, the development of blue InP QLEDs has been slower. This is mainly due to two factors: (1) the low PLQY of QD-assembled light-emitting films and (2) the low carrier injection and transport processes. To address these challenges, several strategies have been proposed. For example, Zhang et al. synthesized thick-shell blue InP/ZnS/ZnS QDs using a wide bandgap ZnS shell to effectively prevent electron transition from the core to the shell and achieved emission from InP QDs at 468 nm. The EQE of these QLEDs was found to reach 1.7% [86]. Du et al. introduced a GaP bridging layer to reduce the lattice mismatch between the InP core and the ZnS shell, resulting in InP/GaP/ZnS quantum dots with thick ZnS shells that exhibited high PLQY (≈81%). QLEDs fabricated using these QDs achieved a L_max_ of 3120 cd m^−2^ and an EQE of 1.01% (Figure 5f) [80]. To improve carrier injection in blue InP QLEDs, Tan et al. introduced an electric dipole layer (EDL) (MoO_3_) with a deep conduction band sandwiched between PEDOT:PSS and PVK to generate voids from PEDOT:PSS. The dipole-induced embedded electric field in the forward direction of the HIL to PVK balances the carrier injection by enhancing hole injection. The p-type doping effect of MoO_3_ increased the carrier concentration of the interfacial PVK and reduced the trap density, thereby increasing its hole mobility. This approach improved the EQE of the blue InP QLED from 1.0% to 2.1% [87]. Based on Tan’s work, Mei et al. developed a strategy for light extraction. Specifically, they extracted light from the waveguide mode to the air mode by using a thin HTL, a high-refractive-index substrate, and substrate surface roughening. The thin HTL and high-refractive-index substrate facilitated the transport of light from the waveguide mode to the substrate mode. The substrate surface was then roughened to further extract light from the enhanced substrate mode into the air mode. This approach improved the EQE of the blue InP QLED from 2.1% to 2.8% and currently represents the highest efficiency for InP blue light emission [6].

The development of InP-based ternary compounds for blue emission has been identified as a significant area of research. In particular, InGaP ternary QDs have emerged as promising candidates for the development of high-quality blue QLEDs [88]. Previous studies have reported on the synthesis of InGaP QDs using two Ga precursors (gallium oleate and gallium acetylacetonate), but the resulting PLQY was low (≈20%) even after the growth of a ZnS shell layer [89]. More recent work by Kim et al. has shown that cation exchange of In^3+^ and Ga^3+^ followed by double growth of the ZnSe inner shell and ZnS outer shell can result in InGaP/ZnSeS/ZnS QDs with tunable blue emission in the 465–475 nm range (depending on the Ga ratio) (Figure 5g). The 465 nm sample showed a high PLQY of 80%, and the corresponding QLED exhibited excellent performance with a L_max_ of 1038 cd m^−2^ and an EQE of 2.5% [81]. The development of InGaP ternary QDs holds great promise for the realization of high-quality blue QLEDs.

## 5. Carbon Dots

### 5.1. Photoluminescence of Carbon Dots

**Figure 6:**
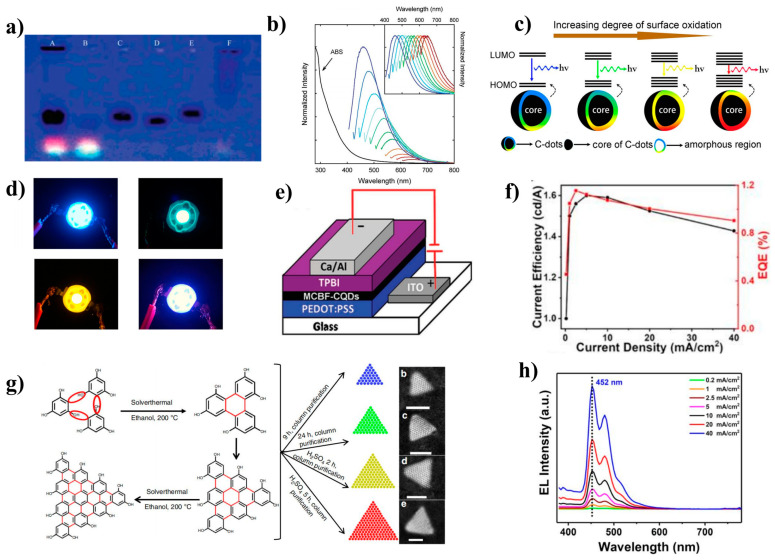
(**a**) Electrophoretic profile in 1% agarose gel under 365 nm UV light [90]. Copyright 2004, American Chemical Society. (**b**) UV–vis absorption and PL spectra of CDs [97]. Copyright 2006, American Chemical Society. (**c**) Model for the tunable PL of CDs with different degrees of oxidation [108]. Copyright 2006, American Chemical Society. (**d**) Optical photographs of blue, green, yellow, and white LEDs [109]. Copyright 2022, Elsevier B.V. (**e**) The device structure of MCBF: CDs-LED [111]. Copyright 2017, Wiley-VCH. (**f**) EQE values (red solid line) and current efficiency (black solid line) of WLEDs [112]. Copyright 2021, Wiley-VCH. (**g**) Synthesis route and HAADF-STEM images of triangular CDs [113]. Copyright 2018, Nature Publishing Group. (**h**) EL emission spectra of the blue CD-LED at different current densities [114]. Copyright 2021, Wiley-VCH.

Carbon dots (CDs) are a type of carbon-based nanomaterial that possess at least one dimension less than 10 nm. The isolation of CDs within single-walled carbon nanotubes (SWNTs) by Xu et al. (Figure 6a) sparked significant interest in their potential applications in optics, leading to numerous studies on their properties [90,91,92,93,94,95,96]. CDs possess a range of advantageous characteristics, such as low cost, low toxicity, good biocompatibility, simple preparation processes, tunable fluorescence emission (Figure 6b), and photochemical stability.

A vast range of raw materials, including laboratory-produced chemicals and natural products, have been used for the synthesis of CDs. This has resulted in a diverse range of components and structures used to prepare CDs, making it difficult to develop a unified theory by comparing the results reported in the literature. The complexity of CDs and the various factors that affect their luminescence processes have also made it challenging to deduce a reasonable PL mechanism. The luminescence of CDs is primarily determined by three characteristics: (1) the material is primarily composed of carbon, (2) carbonization or cross-linking processes are needed, and (3) the kernel luminescence is a carbon-based structure, which is the sp^2^/sp^3^ conjugated structure [97,98,99]. At present, the PL mechanism of CDs is mainly summarized in four aspects: (1) the QCE or conjugate π domain determined by the carbon core [100]; (2) the surface state, which is determined by the hybridization of the carbon backbone and the linked chemical groups; and (3) the molecular state, which is determined only by the fluorescent molecules attached to the surface or interior of CDs; and (4) the crosslinked enhanced emission (CEE) effect [101].

Yan et al. synthesized large-scale colloidal graphene QDs with uniform and tunable sizes through a solubilization strategy [102]. The oxidation of polyphenylene dendritic precursors synthesized by stepwise solution chemistry results in fused graphene moieties, which are stabilized through multiple 2’,4’,6′-trialkyl phenyl groups covalently attached to the edges of the graphene moieties. The bandgap of graphene could be further reduced by increasing its size or chemically functionalizing it to tune its redox potential. In some cases, heteroatom doping can have a more significant effect on the wavelength modulation of CDs than particle size. Yang et al. investigated the use of nitrogen-based precursors (DMF, urea, ethanamide, and formamide) in a solvent-free reaction to achieve multicolor (505–650 nm) fluorescence emission. They revealed that the fluorescence properties of CDs were dependent not only on their particle size but also on their degree of graphitization. The content of C-O and C=N functional groups on the surface of CDs played a synergistic role in constructing multicolor fluorescent CDs [103].

In the absence of group modification, blue-emitting CDs are more easily attainable than longer wavelength-emitting CDs [104,105,106]. However, this does not necessarily imply that the performance of the blue CD-LED is better. The aggregation-induced quenching (ACQ) phenomenon can cause a significant decrease in the fluorescence intensity of CDs in the solid state, leading to a serious change in the spectral characteristics [107]. Ding et al. gradually tuned CDs from blue to red with bright and stable light under the excitation of single-wavelength UV light by silica gel column chromatography [108]. These samples exhibited similar particle size distributions and graphite structures in their carbon nuclei, but their surface states changed gradually. The authors found that the incorporation of oxygen into the surface structure of CDs led to a decrease in the bandgap of these CDs (Figure 6c), resulting in a redshift of the emission peak from 440 to 625 nm.

Recently, Cao’s group confirmed two precise fluorophore structures, DAP and AHP, in o-phenylenediamine (O-PD) CDs [109]. They also extracted two green compounds (G-CD, G-CD 1, and G-CD 2) and one blue compound (B-CD). DAP, G-CD, and B-CD were mixed with polyvinylpyrrolidone (PVP) powder and ethanol to produce a white LED with a color rendering coefficient of up to 87 (Figure 6d). In a study conducted by Yang’s group in 2014, branched polyethylene-imine (PEI) was utilized as a model system to synthesize carbon dots (CDs) by crosslinking with carbon tetrachloride [110]. As a result, the PL of the CDs was found to be significantly enhanced, which was attributed to the reduced vibration and rotation of PEI. This study demonstrated the first instance of cross-linked enhanced emission at the nonconjugated polymerization point.

### 5.2. Electroluminescence of Carbon Dots

CD electroluminescent devices have a more complex luminescence mechanism than inorganic QDs. The device structure of CD-LEDs is similar to that of inorganic QLEDs, such as ITO/PEDOT:PSS/EML/TPBi/LiF/Al (Figure 6e) [111,114,115]. The largest difference lies in the EML. If CD materials are prepared as the EML of the device alone, the luminescence will be low due to aggregation fluorescence quenching. To overcome this issue, CDs are usually incorporated into host materials (such as PVK, CBP, poly-TPD, etc.) to serve as the EML [113,116,117,118]. Host materials not only improve the dispersion of CDs and reduce aggregation fluorescence quenching but also have wider bandgaps that facilitate Förster resonance energy transfer to excite CD luminescence. For example, Wang et al. demonstrated the first preparation of a white CD-LED, which obtained only 0.083% EQE. However, when PVK was used as the host material, the EQE of the white light device was improved by up to 1.18% (Figure 6f) [112,119]. Additionally, Kwon et al. achieved energy transfer from the CBP host to the GQD guest [100]. The energy levels of graphene QDs are located inside the CBP, so charge carriers are injected into the CBP and then transferred from the CBP to the GQD, leading to valuable EL.

Moreover, CDs usually have a broad full width at half maximum (FWHM) emission spectrum due to their structure and surface defects. However, Yuan et al. designed triangular-shaped CQDs (T-CQDs) (Figure 6g) through structural engineering, which greatly weakened electron-phonon coupling and reduced surface defects, resulting in negligible trap-state free exciton emission [113]. These triangular CQDs achieved narrow-bandwidth emission from blue to red (FWHM = 30 nm), with a PLQY of 54–72%, L_max_ = 4762 cd m^−2^, and current efficiency (CE) = 5.11 cd A^−1^.

Furthermore, Sargent’s group explored the effect of oxygen-containing functional groups on spectral broadening using density functional theory [120]. They found that electron coupling to molecular vibration and distortion is the reason for the wide emission spectra. The bandgap vibrations of COOH-CD and NH_2_-CD were compared, and due to the strong polarization effect caused by the COOH functional group, its rotation relative to the base plane affects the positioning degree of the wave function, resulting in the broadening of the spectra.

### 5.3. Blue Carbon Dot Light-Emitting Diodes

The performance of blue CD-LEDs is currently not as good as that of inorganic QLEDs in terms of device luminance, lifetime, EQE, and color purity due to the intrinsic properties of CD materials [117,121]. However, recent research has shown promising results. In 2019, Sargent’s group reported the synthesis of excellent blue CDs using a solution method with citric acid and diaminonaphthalene, resulting in blue CD-LEDs with a L_max_ of 5240 cd m^−2^ and an EQE of 4.04% [120]. Passivating the surface of the CDs with diaminonaphthalene reduced the trap state and increased the PLQY to 70% ± 10%, while decreasing the proportion of oxygen-containing functional groups led to a significant reduction in the FWHM (35 nm). The wide spectra of CD devices have been a significant obstacle in the development of monochromatic devices. However, in 2021, Kang et al. synthesized strongly blue-emitting O and N codoped CDs by the hydrothermal method from perylene-3,4,9,10-tetracarboxylic dianhydride (PTCDA) and 2,3-diamino phenazine (DAP) [119]. The incorporation of these CDs into PVK to form the EML resulted in CD-LEDs emitting at 452 nm, which closely matches the CIE coordinates of the standard pure blue color (0.14, 0.08) specified in NTSC 1953, achieving a purer blue LED (Figure 6h).

## 6. Perovskite QDs

The luminescence properties of perovskite materials vary greatly in different dimensions. Unlike conventional two-dimensional materials that require only a few atomic layers thin to exhibit QCE, perovskite materials can form a natural quantum well structure even with two-dimensional layers due to the isolation of the perovskite layer by long-chain molecular groups. However, the quantum effect of perovskite material depends heavily on the thickness of the perovskite in each layer. While three-dimensional perovskites have high electron mobility, their low film coverage and high defect density have hindered the preparation of high-efficiency devices. Quasitwo-dimensional states lie between two and three dimensions and are a combination of polyphase three- and two-dimensional perovskite layers. Several studies have shown that quasitwo-dimensional structures can combine the advantages of both two- and three-dimensional perovskites, but there remain significant challenges in their development.

PQDs are a type of zero-dimensional perovskite material that exhibits QCE and quantization of near band-edge states when their size is smaller than the Bohr radius. PQDs, similar to other inorganic QDs such as CdSe, InP, and ZnSeTe, exhibit a high PLQY, a narrow emission linewidth, and controllable growth regulation. Two primary methods for achieving deep blue emission in PQDs have emerged. The first approach involves adjusting the size of the PQDs using the ligand-assisted reprecipitation process to control QCE, as demonstrated by Rogach et al. in 2015. By manipulating the temperature, they modulated the emission spectrum of CH_3_NH_3_PbBr_3_ QDs (1.8–3.6 nm) from 475 nm to 520 nm [122]. The second approach involves introducing Cl to modulate the emission wavelength by controlling the ratio of Br/Cl halogens. Kovalenko et al. synthesized CsPbX_3_ and achieved emission spectra throughout the visible region of 410–700 nm by changing the halogen ratio [123,124]. The resulting CsPbX_3_ QDs displayed narrow emission line widths of 12–42 nm, a wide gamut of up to 140% of the NTSC color standard, and a high PLQY of up to 90%.

### 6.1. Quantum Confinement Effect of Perovskite QDs

**Figure 7:**
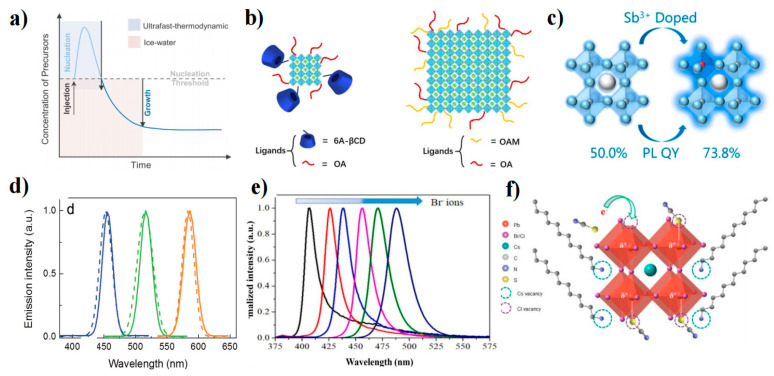
(**a**) La Mer model of PQD growth guidance in IW and UFTDC cooling processes [117]. Copyright 2021, American Chemical Society. (**b**) Growth schematic for ultrasmall CsPbBr_3_ QDs and large CsPbBr_3_ nanocubes [118]. Copyright 2021, Wiley-VCH. (**c**) Schematic of the PLQY change after Sb^3+^ doping [129]. Copyright 2019, American Chemical Society. (**d**) EL spectra (straight line) and PL spectra (dashed line) of CsPbX_3_ QDs [131]. Copyright 2015, Wiley-VCH. (**e**) Normalized PL spectra of CsPbCl_3−x_Br_x_ (x = 0.0–2.5) QDs [132]. Copyright 2021, Elsevier B.V. (**f**) Illustration of Cl^−^ vacancy-induced Coulomb trap site formation, electron trapping, and self-assembly of organic thiocyanate (RSCN) on the defect sites in MHP [133]. Copyright 2020, American Chemical Society.

One approach to achieving spectral modulation in PQD technology is to adjust the bandgap of the PQDs through QCE. This method requires precise control of the crystal growth of the PQDs, which ideally should be close to the Bohr radius. Yang et al. attempted to enhance size uniformity and reduce crystal defects by using ice water and liquid nitrogen to temporarily halt QD growth during synthesis [125]. Ultrafast thermodynamically controlled QDs (UFTDC-QDs) were synthesized using this method and were found to reduce the injection barrier compared to ice water and liquid nitrogen treatment as a control variable for UFTDC (Figure 7a). Perovskite LED (PeLED) prepared on this basis achieved an EQE of 3.66%, a L_max_ of 2100 cd m^−2^ at 460 nm, and a T_50_ of 288 s.

Temperature is not the only factor that can control the reaction process. Mathews et al. used octyl phosphonic acid (OPA) ligands to modulate the growth rate of PQDs [126]. This approach effectively decoupled the PQD nucleation and growth stages, and the reaction duration was determined by preventing PQD growth with the addition of the dodecyl dimethyl ammonium bromide (DDAB) ligand. This method achieved CsPbBr_3_ QD emission wavelengths between 501 and 517 nm. Pradhan et al. reported on CsPbBr_3_ QDs with adjustable wide window sizes [127]. The size and shape of the QDs could be precisely controlled by changing the concentration of the alkylammonium bromide reagent without altering the reaction temperature or the ligand. Cao’s group also investigated the use of macromolecule (6-amino-6-deoxy) beta-cyclodextrin (6A-βCD) as a new cationic ligand to replace the OAM [128]. The spatial hindrance and cage junction of 6A-βCD effectively limited the size of CsPbBr_3_ QDs in three dimensions, achieving blue emission of 400–500 nm (Figure 7b), with a PLQY up to 72.4%.

In addition to the use of large volumes of organic ligands to inhibit PQD growth during the growth process, adjusting the growth environment of the crystal is another approach. Yu et al. found that Sb^3+^ inhibited the further growth of ultrasmall CsPbBr_3_ QDs (2.2–2.9 nm) [129]. The doping of Sb^3+^ reduced the surface energy, improved the lattice energy, passivated defect states, and increased the PLQY to 73.8% (Figure 7c). Zhang et al. designed a polymer gel network by irradiating acrylamide monomers in dimethyl sulfoxide under UV light [130]. The polymer gel controlled the concentration of supersaturated ions and extended the crystallization time to form 1.1 ± 0.2 nm CsPbBr_3_ QDs. Due to excellent surface passivation and QCE, the CsPbBr_3_ QDs achieved a spectrum ranging from 433 nm to 512 nm. The obtained PQDs can emit high-quality blue light with an FWHM of 14 nm and a PLQY of 74%.

### 6.2. Blue Perovskite QDs of Mixed Halogens

The high surface area to volume ratio of small-sized blue PQDs results in a large number of Br- vacancy (VBr) defects on their crystal surface, which adversely affects their optical properties. Achieving efficient deep blue light emission from pure bromine chalcogenides is therefore challenging. To address this issue, a compositional modality of mixing halide (Cl/Br) perovskite has been employed to widen the band gap in the blue spectral region. The Cl/Br ratio can be easily adjusted to tune the band gap, leading to improved optical properties.

Zeng et al. reported the first QLED based on all-inorganic (CsPbX_3_, X = Cl, Br, and I) PQDs, achieving broad spectra from orange to blue (Figure 7d) [131]. Similarly, He et al. synthesized CsPbCl_3−x_Br_x_ (x = 0.0–2.5) QDs using a modified ligand-assisted reprecipitation method and obtained blue emissions with different Br compositions by changing the PbCl_2_ to PbBr_2_ ratio in the precursor [132]. The value of x can be adjusted to precisely control the band gap of CsPbCl_3-x_Br_x_ (x = 0.0–2.5) QDs between 2.54 and 3.06 eV (Figure 7e). However, changes in Cl content can result in changes in halide vacancies and energy states, leading to unbalanced charge injection and an increase in nonradiative sites, causing significant efficiency roll-off. Cl^−^ vacancies are the primary source of mixed halide (Br/Cl) PeLED trap states. To address this issue, we developed a strategy for the passivation of Cl^−^ vacancies in CsPb(Br_x_Cl_1−x_)_3_ QDs using a nonpolar solvent-soluble organic halide [N-dodecyl ammonium thiocyanate (DAT)] (Figure 7f) [133]. Density functional theory calculations showed that the SCN group filled the Cl^−^ vacancy, removing the electron trap in the bandgap, resulting in a stable (~470 nm) and highly efficient (EQE = 6.3%) blue PeLED.

The long chains of insulating ligands hinder the radiative recombination of charges in QDs, reducing the efficiency of PeLEDs. To overcome this limitation, Kovalenko and Kim et al. utilized di-dodecyl dimethylammonium halides (DDAX) as short ligands, which enabled the PQDs to self-reconstruct into a stable formation by surface passivation from OA or OAM to DDA^+^. Surface passivation using DDAX significantly increased the PLQY of PQDs to 50.2% in the deep blue light region at 467 nm [124,134].

## 7. Conclusions and Outlook

QLEDs have experienced unprecedented advances as a new generation of light-emitting components, especially for red and green QLEDs. However, efficient blue QLEDs remain a challenge. In this review, we summarized the development of II-VI (CdSe, ZnSe) QDs, III-V (InP) QDs, CDs, and PQDs (mainly CsPbX_3_) in blue light devices.

Cd-based QDs with great success in achieving high-performance LEDs have inspired researchers to develop solution synthesis of other types of inorganic QDs, with a particular focus on heavy metal-free QDs. The experience gained from the synthesis, ligand modifications, core-shell engineering and device preparation of Cd-based QDs provides a reference for the development of other heavy metal-free QDs.

ZnSe-based QDs have advantages in achieving blue light emission due to their large band gap. In addition to exploiting the QCE, spectral tuning can be achieved by doping Te elements. This offers ZnSe-based QDs a large scope for emission tuning from violet to green. However, the doping of Te significantly broadens the FWHM and makes the spectrum asymmetric. Therefore, achieving high EQE and high color purity will be the next step for blue ZnSe-based QLEDs.

Blue InP QDs face a challenge in achieving deep blue light emission (<470 nm) due to their small core size (<2 nm). The current InP blue QLED efficiency is below 3%. InP can emit in the deep blue range by introducing Ga elements. InGaP QLEDs were reported to emit at 469 nm with an EQE of 2.5%. It is foreseeable that ternary InGaP is the direction of InP-based QD blue light.

From the display perspective, the ACQ effect limits CD luminescence in the solid state, which can only be solved by synthesizing anti-burst CDs and doping them into host materials. Although CD-LEDs are not currently comparable to inorganic QLEDs, CD luminescence has the potential for developing bioimaging applications due to its nontoxic and biocompatible characteristics.

Perovskite QDs have a peculiar crystal structure that enables efficient luminescence. Multicolor luminescence can be achieved by the QCE and modulation of halogen composition. However, deep blue PQDs usually require Br/Cl codoping, which leads to phase separation due to the migration of halogen ions under an electric field. Although several strategies have been proposed to improve PQDs, such as adjusting the halogen ratio, A^-^ and B^-^site doping, and chemical environment regulation, structural instability and poor lifetime remain challenges that PQDs must overcome.

Here, we discussed four representative QDs in material synthesis, core-shell structures, ligand interactions, and device fabrication. We hope to introduce a variety of representative QD materials to help readers understand the problems and solutions encountered in the development of blue QLEDs. As RGB’s most important and difficult part, blue light requires more researchers’ continued attention and efforts.

## Figures and Tables

**Table 1:** Device performance summary of the representative blue CdSe-based QLEDs.

QD	PLQY	Device Performance					
		λ (nm)	EQE (%)	Luminance (cd m^−2^)	CIE (x, y)	Device Structure	Ref
CdZnSe/ZnSeS/ZnS/CdZnS	77.3%	476	8.40%	12,000	(0.11, 0.13)	ITO/PEDOT:PSS/TFB/QD/ZnMgO/Al	[26]
CdSe/ZnSe	73%		8.05%	62,600		ITO/PEDOT:PSS/TFB/QD/ZnO/Al	[35]
CdSe/ZnS	87%	468	19.80%	4890	(0.136, 0.078)	ITO/PEDOT:PSS/PVK/QD/ZnO/Al	[28]
CdZnS/ZnS	82%	448	12.40%	3694	(0.152, 0.024)	ITO/PEDOT:PSS/PVK/QD/ZnO/Al	[36]
CdSe/ZnS		455	10.70%	≈5000	(0.16, 0.02)	ITO/PEDOT:PSS/PVK/QD/ZnO/Al	[38]
ZnCdS/ZnS	70%	443	12.20%	7600	(0.14, 0.02)	ITO/PEDOT:PSS/TFB/QD/ZnO/Al	[39]
Zn_x_Cd_1−x_S/ZnS	100%	445	3.80%	4100		ITO/TFB/QD/ZnO/Al	[37]
CdZnS/ZnS	98%	452	7.1%	2624	(0.153, 0.027)	ITO/PEDOT:PSS/PVK/QD/ZnO/Al	[40]
Cd_1−x_Zn_x_S/ZnS		437	1.7%	2250	(0.17, 0.02)	ITO/PEDOT:PSS/ploy-TPD/QD/TPBi/LiF/Al	[41]
CdSe/ZnS		470	0.22%	4200		ITO/PEDOT:PSS/ploy-TPD/QD/ZnO/Al	[29]
ZnCdS/Cd_x_Zn_1−x_S/ZnS	100%	445	18%	6768		ITO/PEDOT:PSS/PVK/QD/ZnO/Al	[43]
ZnCdSe/ZnS//ZnS	92%	479	16.2%	14,100	(0.119, 0.154)	ITO/PEDOT:PSS/TFB/QD/PMMA/ZnO/Al	[44]

**Table 2:** Device performance summary of the representative blue ZnSe-based QLEDs.

QD	PLQY	Device Performance					
		λ (nm)	EQE (%)	Luminance (cd m^−2^)	CIE (x, y)	Device Structure	Ref
ZnSeTe/ZnSe/ZnS	93%	455	18.60%	12,654	(0.128, 0.109)	ITO/PEDOT:PSS/PVK/QD/ZnMgO/Al	[54]
ZnSe/ZnS	95%	443	13.60%	1031	(0.17, 0.03)	ITO/PEDOT:PSS/PVK/QD/Zn_1-x_Sn_x_O/Al	[55]
ZnSe/ZnS	95%	445	12.20%	570	(0.16, 0.03)	ITO/PEDOT:PSS/PVK/QD/ZnMgO/Al	[65]
ZnSe/ZnS	55%	434	6.88%	450	(0.166, 0.013)	ITO/PEDOT:PSS/PVK/QD/ZnMgO/Al	[56]
ZnSe/ZnS/ZnS	56%	446	2.62%	106	(0.16, 0.02)	ITO/ZnO/QD/CBP/HAT-CN/Al	[66]
ZnSeTe/ZnSe/ZnSeS/ZnS	84%	445	9.50%	2904	(0.148, 0.048)	ITO/PEDOT:PSS/PVK/QD/m-ZnMgO/Al	[58]
ZnSeTe/ZnSe/ZnS	100%	460	20.20%	88,900		ITO/PEDOT:PSS/TFB/QD Cl(f)/Cl(l)/ZnMgO/Al	[10]
ZnSeTe/ZnSe/ZnS	70%	441	4.2%	1195	(0.153, 0.027)	ITO/PEDOT:PSS/PVK/QD/ZnMgO/Al	[53]
ZnSe/ZnS	48%	430	7.83%	2250	(0.169, 0.023)	ITO/PEDOT:PSS/PVK/QD/ZnO/Al	[67]
ZnSe/ZnS		441		1170	(0.16, 0.15)	Al/MoO3/TCTA/CBP/QD/ZnO/ITO	[68]
ZnSe/ZnS	40%	425	0.65%			ITO/PEDOT:PSS/PVK/QD/ZnO/Al	[57]

## Data Availability

Not applicable.
